# Distinctive seizure signature in the first video case-control study of a naturally-occurring feline autoimmune encephalitis model

**DOI:** 10.1016/j.bbi.2025.02.018

**Published:** 2025-05

**Authors:** S.N.M. Binks, A.H. Crawford, E. Ives, L.J. Davison, A. Fower, H. Fox, A. Kaczmarska, M. Woodhall, P. Waters, A.E. Handel, S.R. Irani, R. Gutierrez Quintana, F.A. Chowdhury, S.H. Eriksson, A. Pakozdy

**Affiliations:** a Oxford Autoimmune Neurology Group, Nuffield Department of Clinical Neurosciences, University of Oxford, John Radcliffe Hospital, Oxford OX3 9DU, UK; b Department of Neurology, John Radcliffe Hospital, Oxford University Hospitals NHS Foundation Trust, Oxford OX3 9DU, UK; c The Royal Veterinary College, Hertfordshire AL9 7TA, UK; d Anderson Moores Veterinary Specialists, Winchester, Hampshire SO21 2LL, UK; e Department of Anatomy, Physiology and Genetics, University of Oxford, Sherrington Building, Sherrington Rd, Oxford OX1 3PT, UK; f Departments of Neurology and Neurosciences, Mayo Clinic, Jacksonville, FL, USA; g University of Glasgow, School of Biodiversity, One Health and Veterinary Medicine, Small Animal Hospital, 464 Bearsden Rd, Glasgow G61 1QH, UK; h Neurology, National Hospital for Neurology and Neurosurgery, London, UK; i Department of Clinical and Experimental Epilepsy, UCL Institute of Neurology, University College London, London, UK; j University Clinic for Small Animals, University of Veterinary Medicine, Vienna, Austria

**Keywords:** Autoimmune, Cats, Encephalitis, Leucine-rich glioma-inactivated 1 (LGI1), Orofacial, Seizures

## Abstract

**Background and objective:**

Autoimmune encephalitis (AE) is a form of brain inflammation where pathogenic autoantibodies bind surface proteins. In humans, AE is at least as common as infective encephalitis, and seizures are a prominent manifestation. The most common adult human AE is associated with antibodies to leucine-rich glioma-inactivated 1 (LGI1-Ab-E). AE in non-human mammals is also recognised, notably the polar bear ‘Knut’, diagnosed with N-methyl D-aspartate receptor antibody encephalitis. LGI1-Ab-E is an emerging cause of spontaneously-arising AE in domestic cats. Our objective was to phenotype the seizure profile of feline LGI1-Ab-E and probe parallels to its human counterpart.

**Methods:**

We characterised seizures in naturally-occurring feline LGI1-Ab-E. Three veterinary and two human neurologists independently blind-rated 35 LGI1-antibody positive and negative feline seizure videos from 24 cats (16 LGI1-Ab-E positive, 8 negative). Data analysed included seizure frequency, semiologies and their co-occurrence, localisation, inter-rater agreement, and predictive factors.

**Results:**

The mean number of daily seizures at peak was significantly higher in LGI1-antibody positive compared to LGI1-antibody-negative cats (12.6 vs. 1.9/day, pcorr = 0.011). Semiologies statistically significantly enriched in LGI1-Ab-E observations included orofacial automatisms (88/120, 73 % vs. 26/55, 47 %, pcorr = 0.024), salivation (87/120, 73 % vs. 23/55, 42 %, pcorr = 0.004); and mydriasis (79/120, 66 % vs 19/55, 35 %, pcorr = 0.004), and almost exclusively seen in LGI1-Ab-E were circling (39/120, 33 % vs. 1/55, 2 %, pcorr=<0.001) and aggression (14/120, 12 % vs. 0/55, 0 %, non significant after correction). A temporal lobe onset was proposed in 67 % (80/120) of seropositive ratings, compared to 28 % (15/55) LGI1-Ab-E negative (p < 0.0001). Network analysis depicted complex and overlapping relationships between features, akin to the frequent and multifaceted seizures of human LGI1-Ab-E. Orofacial automatisms, mydriasis and temporal lobe localisation were predictive semiological features of feline LGI1-Ab-E.

**Significance:**

Feline LGI1-Ab-E represents a clinically distinctive seizure disorder. Our findings highlight the value of studying naturally-occurring, biologically representative animal models which closely mimic human diseases. This bidirectional translational approach confers benefits across species and unites human and veterinary neurology.

## Highlights

• Leucine-rich glioma-inactivated 1-antibody encephalitis (LGI1-Ab-E) occurs in cats.
• Akin to human LGI1-Ab-E, it presents an explosive-onset focal seizure disorder.
• Characteristic semiologies include orofacial automatisms, salivation, and mydriasis.
• Focal seizures in feline and human LGI1-Ab-E mostly arise from the temporal lobe.
• Our ‘One Health’ approach has translational potential and unites disciplines.

## 1 Introduction

Autoimmune encephalitis (AE) is a form of brain inflammation, in which pathogenic antibodies bind surface proteins and modulate their physiological actions, typically causing clinical manifestations of seizures, cognitive deficits and behavioural change. (Leypoldt et al., 2015, Prüss, 2021) In humans, AE is at least as common as infective encephalitis, and known as an important cause of reversible central nervous system dysfunction, treatable with immunotherapy. (Dubey et al., 2018, Thompson et al., 2018) AE in non-human mammals is recognised, notably in the Berlin Zoo polar bear ‘Knut’, who had N-methyl D-aspartate receptor antibody encephalitis (NMDAR-Ab-E), discovered on post-mortem histopathology after he drowned during a seizure in 2011 (Prüss et al., 2015).

The most common adult human AE is associated with antibodies to leucine-rich glioma-inactivated 1 (LGI1-Ab-E). (Zuliani et al., 2021, Kunchok et al., 2022) Since LGI1 is richly expressed in the hippocampus, (Chernova et al., 1998, Kalachikov et al., 2002) a highly epileptogenic brain region, (Chowdhury et al., 2021) the pathophysiological signature of human LGI1-Ab-E is hippocampal inflammation, (Irani et al., 2010) and its clinical signature, an acute-onset seizure disorder. EEG studies in human LGI1-Ab-E have demonstrated frequent temporal lobe-onset clinical and sub-clinical seizures displaying multiple semiologies. (Aurangzeb et al., 2017, Steriade et al., 2016) In addition, around 60 % of human LGI1-Ab-E patients experience a pathognomonic and instantly recognisable seizure type, faciobrachial dystonic seizures (FBDS), consisting of brief dystonic posturing of the hemi-arm, face and/or leg. (Thompson et al., 2018, Irani et al., 2011, van Sonderen et al., 2016).

LGI1-Ab-E is emerging as a cause of spontaneously-arising AE and acute-onset seizures in domestic (pet) cats. Four feline cases were first described in 2013, (Pakozdy et al., 2013) with a further 26 reported in 2023. (Glantschnigg-Eisl et al., 2023) These cats present with new seizures, and investigations reveal comparable imaging, electrographic and neuropathological findings to their human counterparts. Moreover, serological testing by cell-based assay (CBA), a routine method in the detection of LGI1-Ab-E, identifies naturally-occurring LGI1-antibodies. Therefore, these feline patients represent a spontaneous animal model of disease with both evolutionary and biological resonance. (Binks et al., 2022).

The International Feline Encephalitis Study Group was established in 2019 to study AE in domestic cats from a cross-disciplinary perspective. We had curated a number of videos, from the in-hospital or home setting, of seizure episodes in cats whose sera we had screened for LGI1-antibodies. This video resource included episodes from LGI1-antibody-positive and −negative cats. The present study sought formally to characterise and compare the seizures in both groups of cats through a video-rating methodology, bringing together human and veterinary neurologists. Here, we aim to rate these videos to define distinctive seizure signatures in naturally-occurring feline LGI1-Ab-E.

## 2 Materials and methods

**Fig. 1:**
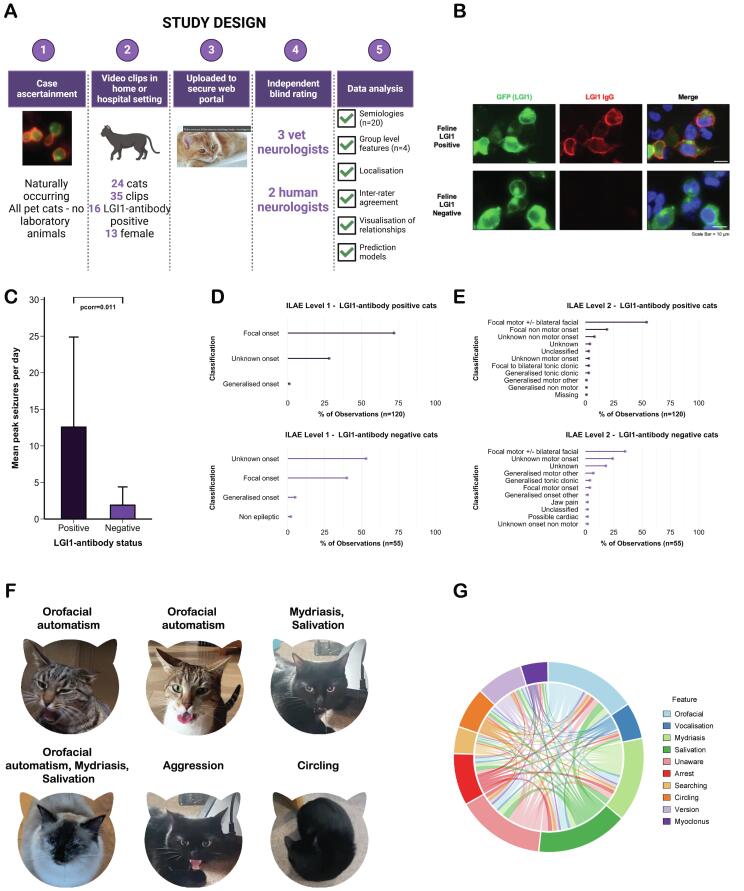
**Experimental plan and results of the first video study of a naturally-occurring feline autoimmune encephalitis model. [A]** Study design **[B]** Cell based assay from a LGI1-antibody-positive cat (top row; Cat 1- titre 1:40) in the study, and a representative negative control cat (bottom row). Left-Right: HEK cells transfected with a feline LGI1 construct tagged with EGFP, with a feline anti-Fc gamma receptor antibody applied after incubation with patient serum at 1:20 dilution, and images merged with the addition of nuclei stained with DAPI (4′,6-diamidino-2-phenylindole). All images taken on a fluorescent microscope at 100 x magnification. Scale bar represents 10 μm **[C]** Bar chart depicting mean peak daily seizure number at onset in LGI1-Ab-E positive (dark purple) compared to LGI1-antibody-negative (light purple) cats. Error bar shows upper limit of standard deviation **[D, E]** Lollipop diagrams showing percent of observations of localisation based on top-level **[D]** and second-level ILAE **[E]** classifications in LGI1-antibody-positive (top, total observations = 120) and −negative (bottom, total observations = 55) clips **[F]** Video stills from study clips showing key features of orofacial automatisms, mydriasis, salivation, aggression, and circling in study cats **[G]** Circos plot delineating connectivity between top 10 observed features in LGI1-Ab-E cats. Each colour segment proportionally represents a semiology, and its co-occurrence with other semiologies is also proportionally represented through incoming or outgoing projections. **Abbreviations:** HEK, human embryonic kidney cells; ILAE, International League Against Epilepsy; LGI1, leucine-rich glioma-inactivated 1; LGI1-Ab-E, LGI1-antibody encephalitis.

A summary of the study design is included in Fig. 1A. This study was carried out under Royal Veterinary College Clinical Research Ethical Review Board approval (URN: 2020 1957-2), and client consent was obtained for inclusion in the research programme. All included cats are domestic (pet) cats. No laboratory animals were used or procedures carried out as part of this study.

### 2.1 Case ascertainment

Included cases were enrolled in a parent study testing cats with epilepsy of unknown aetiology for LGI1-antibodies. Sera submitted by treating veterinarians were screened on a live CBA in Oxford, as previously described in cats and humans. (Thompson et al., 2018, Irani et al., 2010, Glantschnigg-Eisl et al., 2023, Michael et al., 2020) In brief, human embryonic kidney (HEK293T) cells in culture were seeded onto 24 well plates and transiently transfected with a feline LGI1 (FEL-LGI1) construct tagged with a green fluorescent protein. After 24 h, feline sera were applied at a starting dilution of 1:20 and incubated for one hour with the FEL-LGI1-expressing cells. After a washing step, cells were lightly fixed with 4 % formaldehyde and a secondary detection antibody was applied (Jackson Immuno Research Alexa Fluor®-594-conjugated AffiniPure Goat Anti-Cat IgG Fcγ Fragment Specific, 102–585-008) at 1:500. All positive sera were titred out to their endpoint on serial dilution doublings. To confirm LGI1 specificity, all sera were additionally screened against a different construct, feline contactin-associated protein-like 2 (CASPR2). Fig. 1B shows example CBAs from a LGI1-antibody-positive and negative case.

### 2.2 Video collection and seizure counts

Videos of seizure episodes were submitted to the study between 2019 and 2023 and had been ecologically captured in the home or hospital setting. Available clips received during this time were included in the video rating study if the LGI1-antibody status of the cat could be verified. Demographic data were provided by submitting veterinarians. Some clips, with additional client consent, were included on a password-protected study microsite for veterinary and medical professionals available at: https://www.rvc.ac.uk/research/feline-encephalitis.

Daily seizure counts were established from clinical review of the submitting veterinarian, augmented by owner-completed retrospective questionnaires in four cats. Peak daily seizures were calculated as maximum episodes per day during disease onset. For cats having less than 1 daily seizure at peak, the number of seizures was divided over the time span given e.g. one every two days = 0.5/day.

### 2.3 Rating

A standardised rating tool (Excel spreadsheet, Supplementary Materials) with pre-defined parameters was devised with reference to core veterinary and human reference documents (Berendt et al., 2015, Fisher et al., 2017, Sato, 1975, Scheffer et al., 2017) and prior video rating methods. (Varley et al., 2019) The final version included 20 specified semiologies grouped under main headings of automatisms, autonomic, awareness, behavioural, motor, and other. These fields were pre-set to yes/no answers to maximise data collection. The rating tool also contained more freeform fields for classification and localisation, guided by International League Against Epilepsy (ILAE), (Fisher et al., 2017, Scheffer et al., 2017) International Veterinary Epilepsy Task Force (IVETF) (Berendt et al., 2015) and Sato staging, (Sato, 1975) with the option of selecting ‘unknown’, if this could not be determined for any reason including seizure onset not captured.

Video clips were uploaded to a secure web portal. To facilitate a comparable approach across raters, guidance was provided on use of the tool and how to approach uncertainty (Supplementary Results and Methods). Clips were grouped randomly as to antibody status. They were presented in a standardised order via the ratings tool, but there was no restriction as to viewing sequence or number of times they could be viewed. Five raters (three veterinary neurologists and two human epileptologists) viewed and rated the clips via the rating tool, independently and blind to the LGI1-antibody status of the cat. None of the veterinary neurologists were involved in the care of the included cats.

### 2.4 Statistical methods and data visualisation

All statistical analyses were performed in R (v4.0.3 and v4.4.0). Between-group comparisons of categorical variables were made with chisq or Fisher’s test (for contingency tables with five or fewer observations in any one group), and *t*-test (normally distributed data) or Wilcoxon’s sum rank test (non-normally distributed data) for continuous variables. Holm adjustment was used for multiple comparison correction. Distribution of continuous data was interrogated by the Shapiro-Wilk normality test. Inter-rater agreement was assessed by Fleiss’ kappa using the IRR (v0.84.1) and DescTools (v0.99.54) packages in R. Visualisation of semiology relationships by circos plot was performed with the R circlize package (v0.4.16). (Gu et al., 2014) Logistic regression and associated statistics were carried out in base R. Significance was set at p < 0.05. Additional data visualisation and graphics were obtained with R ggplot2 and cowplot (v 1.1.3), and with Biorender.

## 3 Results

### 3.1 Included cats and videos

Thirty-five videos were available from 24 cats (16 LGI1-Ab-E positive, 8 LGI1-Ab-E negative; 13 female and 11 male) from the United Kingdom, Belgium and Italy, with seven cats (five LGI1-antibody positive, two LGI1-antibody negative) contributing more than one video (Supplementary Results Table 1). Therefore, in the primary analysis, there was a total of 120 observations per feature (five raters by 24 clips) in the LGI1-Ab-E group and 55 observations per feature (five raters by 11 clips) in the LGI1-antibody-negative group. Post-hoc sensitivity analyses were also performed, restricted to one clip per cat. The video with the longest footage was chosen, yielding a total of 80 (five raters by 16 clips) in the LGI1-Ab-E group and 40 (five raters by 8 clips) in the antibody-negative group (Supplementary Results Table 2). There was no difference in sex distribution, mean seizure onset age or proportion of domestic short or long hair (hence, referred to as DSH and DLH) cats in the LGI1-antibody-positive or −negative groups (Table 1).

**Table 1:** Features of included cats and video clips.

	**LGI1-antibody positive**	**LGI1-antibody negative**	**p-value^a^**
**Participant contributions**			
*Number of patients*	16	8	n/a
*Number of clips*	24	11	n/a
*Total seconds of footage*	1183	420	n/a
*Total observations per sign*	120	55	n/a
**Participant demographics**			
*Sex / neuter (N) status*	8 FN, 8 MN	5F (4 FN), 3 M (2 MN)	1^b^
*Mean onset age / months (median, range)*	47 (49.5, 17–96)	37 (22.5, 4–110)	1^c^
*Breeds*	11 Domestic Short/Long Hair, 2 Bengal, 1 each British Short Hair, Exotic, Ragdoll	4 Domestic Short Hair, 1 each Bengal, Birman, British Short Hair, Turkish	1^d^
*Country of origin*	UK (13), Belgium (2), Italy (1)	UK (6), Belgium (1), Netherlands (1)	n/a
*Median antibody titre (range)*	120 (20–320)	negative	n/a
*Mean peak number seizures per day at onset (median, range)*	12.6 (7.5, 3–48)	1.9 (0.07, 0–6)	0.011^e^

^a^ Holm corrected for multiple comparisons ^b^Fisher’s exact test, proportion male/female in each group ^c^unpaired *t*-test ^d^Fisher’s exact test, proportion domestic short/long hair in each group ^e^Wilcoxon sum rank test. Data on peak number of seizures/day at onset were missing for 2 seropositive and 1 seronegative cat.

Abbreviations: F, female; FN, female neutered; M, male; MN, male neutered.

### 3.2 Observed features

Firstly, the mean number of estimated daily seizures at peak was significantly higher in the LGI1-Ab-E compared to the LGI1-antibody-negative group (12.6 vs. 1.9/day, pcorr = 0.011, uncorrected p = 0.003) (Table 1 and Fig. 1C). Next, we counted the number of times the five raters judged a pre-specified feature as present in LGI1-antibody-positive (total observations-per-feature, 120) compared to LGI1-antibody-negative (total observations-per-feature, 55) cats. The top four features observed in LGI1-Ab-E cats were orofacial automatisms (88/120, 73 % of observations), salivation (87/120, 73 % of observations), reduced awareness (81/120, 68 % of observations), and mydriasis (79/120, 66 % of observations). Compared to LGI1-antibody-negative cats, orofacial automatisms (73 % vs. 47 % (26/55), pcorr = 0.024), salivation (73 % vs. 42 % (23/55), pcorr = 0.004), and mydriasis (66 % vs. 35 % (19/55), pcorr = 0.004) were all significantly enriched in the antibody-positive group. Circling was also characteristic, being identified in 39/120 (33 %) LGI1-Ab-E positive compared to 1/55 (2 %) LGI1-antibody-negative clips (pcorr < 0.001). Aggression was only depicted in the LGI1-antibody-positive cats (14/120, 12 %), but this was not significant after correction for multiple comparisons. Therefore, on a per-observation basis, four features were significantly enriched and distinctive for the LGI1-Ab-E cats (Table 2, Fig. 1F and Supplementary Online Figure).

**Table 2:** Observed features in LGI1-antibody positive compared to LGI1-antibody negative cats.

**Feature**	**LGI1-antibody positive (total observations n = 120)**	**LGI1-antibody negative (total observations n = 55)**	**p-value raw**^a^	**p-value corrected**^b^
**A: Individual observations**				
**Automatisms**				
*Orofacial*	88 (73 %)	26 (47 %)	0.001	0.024
*Running/pedal*	12 (10 %)	7 (13 %)	0.782	1
*Vocalisation*	26 (22 %)	3 (5 %)	0.008	0.109
**Autonomic features**				
*Mydriasis*	79 (66 %)	19 (35 %)	<0.001	0.004
*Respiratory changes*	14 (12 %)	10 (18%)	0.3542	1
*Salivation*	87 (73 %)	23 (42 %)	<0.001	0.004
*Urination/defecation*	1 (<1%)	6 (11 %)	0.004	0.07
**Awareness**				
*Reduced responsiveness*	81 (68 %)	31 (56 %)	0.209	1
**Behavioural features**				
*Aggression*	14 (12 %)	0 (0 %)	0.006	0.084
*Behavioural arrest*	45 (38 %)	15 (27 %)	0.250	1
*Fearful*	10 (8 %)	1 (2 %)	0.177	1
*Restless/searching*	22 (18 %)	7 (13 %)	0.480	1
**Motor features**				
*Circling*	39 (33 %)	1 (2 %)	<0.001	<0.001
*Head nodding*	26 (22 %)	16 (29 %)	0.381	1
*Head turning/ nodding/version*	35 (29 %)	18 (33 %)	0.765	1
*Myoclonus*	21 (18 %)	14 (25 %)	0.309	1
*Sudden jumping*	11 (9 %)	6 (11 %)	0.931	1
*Tonic-clonic jerking*	17 (14 %)	15 (27 %)	0.061	0.674
*Tonic paw extension*	19 (16 %)	18 (33 %)	0.019	0.250
**Other**				
*GTCS*	10 (8 %)	12 (22 %)	0.024	0.292
**B: Group level observations**				
*Any automatism*	92 (77 %)	28 (51 %)	0.001	0.004
*Any autonomic*	105 (88 %)	34 (62 %)	<0.001	<0.001
*Any behavioural*	68 (57 %)	19 (35 %)	0.011	0.021
*Any motor*	87 (73 %)	45 (82 %)	0.254	0.254
**C: Localisation**				
*Temporal lobe*	80 (67 %)	15 (28 %)^c^	<0.001	n/a

^a^Chisq test (if > 5 in all groups) or Fisher’s exact test. ^b^Holm corrected for within-group multiple comparisons, corrected p < 0.05 taken as significant. ^c^One missing observation, total n = 54.

**Abbreviations:** GTCS, generalised tonic clonic seizure.

We also interrogated whether an episode exhibiting at least one feature from each of the four main categories (automatism, autonomic, behavioural, or motor, as described in Table 2A) was indicative of LGI1-antibody positivity. Overall, seizures having at least one automatism, autonomic, or behavioural semiology were significantly more likely to belong to the LGI1-antibody positive than −negative group, whereas motor features did not differentiate between the two (Table 2B).

A similar picture was seen in the one-clip-per cat analysis. Although this included fewer observations, mydriasis (53/80 (66 %) vs. 11/40 (27.5 %) pcorr = 0.003) and circling (23/80 (29 %) vs. 1/40 (2.5 %) (pcorr = 0.009) remained significantly enriched in LGI1-antibody cats after multiple comparison correction, while salivation (55/80 (69 %) vs. 19/40 (47 %) was significant on raw p-value (uncorrected p = 0.04) (Supplementary Table 2). Orofacial automatisms were no longer significantly enriched, but per-cat analysis showed that these were differentially observed in 6/7 cats with multiple clips, pointing to a loss of varied semiology as a potential reason. Tonic paw extension, which was not significant after multiple comparison correction in the primary analysis, was retained as more frequent in the LGI1-antibody negative cats (17/40, 42.5 % vs. 12/80, 15 %, pcorr = 0.036).

### 3.3 Localisation and classification

In humans with LGI1-Ab-E, many focal seizures arise from the temporal lobes. (Aurangzeb et al., 2017) Therefore, we assessed whether a temporal onset was judged more likely in clips of LGI1-antibody associated seizures compared to those from the LGI1-antibody-negative group. The expert raters suggested a temporal lobe origin in 80/120 (67 %) observations of LGI1-Ab-E cats, whereas only in 15/54 (28 %(p < 0.001)) non-LGI1-antibody associated observations (one missing rating for a negative cat). Thus, feline LGI1-Ab-E, like its human counterpart, significantly associates with more clinically-judged temporal lobe seizures (Table 2C). This was also true of the supplementary analysis (Supplementary Table 2).

We also classified seizures in line with the ILAE 2017 and the IVETF 2015 guidelines (Fig. 1D and E and Supplementary Results Table 3). The majority of seizure observations from LGI1-Ab-E cats were classified as focal onset (86/120, 72 %), and on next-level classification as either focal motor onset with or without bilateral facial involvement (65/120, 54 %) or focal non-motor onset (23/120, 19 %). By contrast, a more heterogenous observational picture emerged in the LGI1-antibody-negative group: 22/55 (40 %) with focal onset and 29/55 (53 %) with unknown onset, with next-level classifications including focal motor onset with or without bilateral facial involvement (19/55, 35 %), unknown motor onset (13/55, 24 %) and unknown (10/55, 18 %). Hence, LGI1-antibody positivity in cats presents a distinctive phenotype of focal seizures with predominant motor onset and facial involvement, as recognised and categorised by internationally agreed rating scales.

### 3.4 Inter-rater agreement

Next, using Fleiss’ kappa statistic, we asked if there was agreement between the five raters for 21 analysed features (Table 3), particularly given our mixed rating by both veterinary and human neurologists. We found at least moderate agreement (Fleiss’ kappa ≥ 0.41) in 8/21 (39 %) and slight to fair agreement in the remaining parameters, although this was not significant for reduced responsiveness. No parameters were judged to have poor agreement.

**Table 3:** Inter-rater agreement calculated by Fleiss’ kappa, with 95% confidence intervals (CI).

**Feature**	**Fleiss’ kappa (95 % CI)**	**pvalue**
**Almost perfect agreement (0.81**–**1)**		
Circling	0.838 (0.733–0.943)	<0.001
**Substantial agreement (0.61**–**0.80)**		
Generalised tonic-clonic seizures	0.766 (0.661–0.871)	<0.001
Urination/defecation	0.702 (0.598–0.807)	<0.001
Tonic-clonic jerking	0.771 (0.666–0.875)	<0.001
**Moderate agreement (0.41**–**0.60)**		
Salivation	0.547 (0.442–0.652)	<0.001
Orofacial automatisms	0.497 (0.391–0.601)	<0.001
Temporal lobe localisation	0.459 (0.354–0.564)^a^	<0.001
Vocalisation	0.442 (0.337–0.547)	<0.001
**Fair agreement (0.21**–**0.40)**		
Mydriasis	0.339 (0.234–0.444)	<0.001
Aggression	0.34 (0.235–0.445)	<0.001
Tonic paw extension	0.297 (0.193–0.402)	<0.001
Sudden jumping	0.283 (0.179–0.388)	<0.001
Respiratory changes	0.276 (0.171–0.380)	<0.001
Restless/searching behaviour	0.256 (0.151–0.361)	<0.001
**Slight agreement (0**–**0.20)**		
Running/pedal automatisms	0.203 (0.098–0.308)	<0.001
Behavioural arrest	0.201 (0.096–0.306)	<0.001
Fearful	0.175 (0.071–0.280)	0.001
Head turning/version/nodding	0.161 (0.056–0.266)	0.003
Myoclonus	0.143 (0.038–0.248)	0.008
Head nodding	0.123 (0.018–0.228)	0.022
Reduced responsiveness	0.008 (−0.010–0.113)	0.882
^a^One missing observation treated as negative		

### 3.5 Relationships between semiologies in LGI1-Ab-E

To examine the co-occurrence of semiologies within LGI1-Ab-E cats we performed visualisation with an adjacency matrix chord diagram (Fig. 1G). We selected the 10 most frequently observed semiologies in LGI1-Ab-E cats, replacing head nodding (as it showed overlap with the head nodding/turning/version category and a lower kappa (0.123 compared to 0.161)) with myoclonus. The diagram depicted a complex phenotype in which the four dominant features (orofacial automatisms, salivation, reduced awareness, and mydriasis) were seen in conjunction with each other and multiple others. This echoed the group-level numeric analysis, in which a majority of LGI1-Ab-E clip observations contained automatisms, autonomic and behavioural components. Moreover, it is comparable to human video studies which have shown multiple semiologies present within the same LGI1-Ab-E patient. (Aurangzeb et al., 2017).

### 3.6 Logistic regression

Finally, we performed logistic regression to explore whether specific semiologies could predict the presence of LGI1-antibodies. We considered a feature to be present if at least three of the five expert raters had observed it per clip and included the 10 most frequently depicted semiologies and localisation. Overall, the presence of orofacial automatisms, mydriasis, and a suspected temporal lobe origin all produced statistically significant individual regressions explaining a substantial proportion of phenotypic variance. Resultant odds ratios to predict LGI1-Ab-E were 6.65 (orofacial automatisms and mydriasis) and 9 (temporal lobe origin; Supplementary Results 4A-C). In contrast, models combining a temporal lobe origin with orofacial automatisms and/or mydriasis were not significant. Sensitivity analyses showed that only temporal lobe origin significantly predicted LGI1-antibody positivity, with an odds ratio of 11, when four of five expert raters agreed its likelihood in a clip (Supplementary Results 4D). Therefore, the presence of clinically-diagnosed temporal lobe origin seizures is the most effective semiological predictor of feline LGI1-Ab-E.

## 4 Discussion

Feline limbic encephalitis or epilepsy displaying focal orofacial seizures in association with LGI1-antibodies was first reported in four cats in 2014, (Pakozdy et al., 2013) and observed in 26 animals in 2023. (Glantschnigg-Eisl et al., 2023) However, we are the first to evidence a distinctive seizure signature in naturally-occurring feline LGI1-Ab-E through a video-rating methodology, and capture in detail its semiologies and expert-assessed localisation. Using a video-rating methodology, we identified seizure frequency, localisation and individual features including orofacial automatisms, salivation, mydriasis, and circling. There are differences to human disease in terms of semiological manifestations, with, for example, drooling and circling not seen in human LGI1-antibody associated seizures. However, the high frequency, variety of seizures and their localisation, is comparable to human LGI1-Ab-E, demonstrating the translational potential of this naturally-occurring feline version. We have equalled patient numbers examined in human-only studies. (Aurangzeb et al., 2017, Steriade et al., 2016) Our novel approach harnesses clinical expertise in both veterinary and human neurologists and highlights the importance of “one species” autoimmune seizures cross-cutting feline and human disease. (Bakpa et al., 2016) Our live CBA focused on LGI1-antibodies, which markedly improves clinical specificity over use of VGKC (voltage gated potassium channel) antibodies, which were studied in early feline cohorts (Pakozdy et al., 2013, Glantschnigg-Eisl et al., 2023, Michael et al., 2020).

Feline LGI1-Ab-E patients in this study had a mean daily seizure tally at onset of 12.6, almost identical to the 12 per day focal events reported in one human cohort. (van Sonderen et al., 2016) The proportion of observations of seizures of temporal lobe origin, at 67 %, mirrors 62 % in a human video EEG study, the gold-standard method in seizure adjudication. (Aurangzeb et al., 2017) However, we did not observe any single seizure type pathognomonic of disease akin to FBDS seen in human LGI1-Ab-E. (Irani et al., 2011) Rather, a constellation of semiologies, localisation, and seizure frequency proved characteristic of feline LGI1-Ab-E and may aid its recognition by veterinary surgeons. As visualised in our co-occurrence matrix, predominant features of orofacial automatisms, mydriasis, salivation, and reduced awareness, interacted in a complex way with multiple other semiologies. This is also reminiscent of the intricacies of the psychopathology and movement disorder of NMDAR-Ab-E, and speaks to autoimmune channelopathies involving varied anatomical sites and connectivities according to their antigenic distribution. (Varley et al., 2019, Al-Diwani et al., 2019) Reasons for FBDS absence in cats remain to be explored. Potential explanations could include comparative neuroanatomy, (Toossi et al., 2021) quadrupedal gait, (Vilensky, 1987) or simply they have as yet gone unobserved. Nevertheless, an intriguing parallel is the rarity of FBDS in paediatric LGI1-Ab-E cohorts. (Nosadini et al., 2019).

The existence of spontaneous temporal lobe epilepsy (TLE) in cats has been debated, (Pakozdy et al., 2023) but was staged experimentally by Sato in 1975. (Sato, 1975) Features consistent with all of Sato’s stages were observed by the expert raters including searching behaviour (Stage 1, 22/120, 18 % observations in LGI1-Ab-E cats), behavioural arrest (Stage 2, 45/120, 38 % observations in LGI1-Ab-E cats), orofacial automatisms (Stage 3–4, 88/120, 73 % observations in LGI1-Ab-E cats), head turning/nodding (Stage 5, 35/120, 29 % observations in LGI1-Ab-E cats) and secondary generalisation (Stage 6, 10/120, 8 % observations in LGI1-Ab-E cats). Therefore, our study also appends additional evidence for feline TLE. The distribution of Stages 1–6 in LGI1-Ab-E cats is compatible with a predominant focal seizure disorder with less frequent generalisation, a pattern also known in human LGI1-Ab-E. (van Sonderen et al., 2016, Gadoth et al., 2017, Smith et al., 2021).

There are limitations to our study. Our footage was organically captured in the home or hospital setting, and lacks EEG correlation, although video EEG in an LGI1-antibody-positive cat was previously reported. (Pakozdy et al., 2014) Contribution of more than one clip per cat could represent a source of bias, although our sensitivity analyses to explore this were in keeping with our primary approach. The principal difference, applying to orofacial automatisms, could be explained by loss of varied intra-cat semiology, as is seen in human patients. We received more LGI1-Ab-E than non LGI1-Ab-E videos, representing a longer total footage time in the LGI1-Ab-E group (Table 1). The clips did not always show offset and onset of the events. As they were not bespoke recordings, some aspects were difficult to ascertain, for example, mydriasis, which could be obscured or confounded by dim lighting. Reduced awareness was difficult to ascertain, reflected by its low kappa rating, and certain features were inherently more subjective, such as aggression and fearfulness. However, this also applies to the clinical setting and overall kappa ratings were encouraging, being comparable to those achieved in a previous study in 15 veterinary surgeons of canine paroxysmal events. (Packer et al., 2015).

The parent study, while established to probe the occurrence of LGI1-antibody-associated and other immune seizure aetiologies in cats, receives samples of all-cause non-infectious new-onset feline seizures. Despite these relatively broad entry criteria, in light of our investigatory focus, the LGI1-antibody negative group could be enriched for suspected autoimmune epilepsy. We cannot rule out an autoimmune cause, potentially with autoantibodies to antigens other than LGI1 or CASPR2, in these cats. While this could, in theory, reduce the sensitivity of our modelling to detect LGI1-Ab-E specific features, this was not the case in EEG-studied human LGI1-Ab-E, in which the control group included other autoimmune patients. (Steriade et al., 2016) Although raters were blinded as to the antibody status of studied cats, they were aware that some of the cats did harbour LGI1-antibodies. It is likely that this focussed video study was not powered to build multivariate logistic regression models. Larger cohorts and datasets will be needed to explore factors predictive of feline LGI1-Ab-E. All cats entered into our study were assessed as having a non-infectious cause of seizures. However, the potential impact of region-specific infectious aetiologies to the applicability of our findings in cohorts from outside Europe remains to be explored.

## 5 Conclusion

Taken together, we show that naturally-occurring feline LGI1-Ab-E mimics the explosive-onset focal seizures characteristic of human disease. Notably, these have been difficult to replicate in laboratory rodent models, (Petit-Pedrol et al., 2018, Ramberger et al., 2020) subtracting from their relevance to human LGI1-Ab-E. Our approach of studying LGI1-Ab-E arising intrinsically in domestic cats as a bidirectional translational model represents a potential route to a shared neurobiological ‘ground truth’. Future directions could implement joint human-feline patient video rating studies, and characterise other relevant phenotypic aspects of LGI1-Ab-E in cats, including cognitive and behavioural impairments, as well as screening in a more general feline epilepsy cohort. Our current study not only has translational potential, but also offers the chance to move away from a purely human-centric to an inclusive ‘One Health’ approach, pooling neurological expertise, and ensuring benefits accrue to all the investigated species (Devinsky et al., 2018).

## Funding statements

**Dr Binks** is currently funded by a NIHR clinical lectureship, and grants from PetPlan (S20-924-963), PetSavers (03:20) and a Wellcome PhD award (102176/Z/13/Z) have funded this work. She currently holds grants from the Morris Animal Foundation (D24FE-810) and PetSavers (MDR 12.22) and is a co-applicant on PetPlan (S23-1266-1305). **Dr Crawford** is a co-applicant on grants from PetPlan (S20-924-963, S23-1266-1305), PetSavers (03:20), PetSavers (MDR 12.22), and the Morris Animal Foundation (D24FE-810). **Dr Ives** is an employee of Anderson Moores Veterinary Specialists. **Professor Davison** has been supported by an MRC Clinician Scientist Fellowship (MR/R007977/1) and an MRC Transition Support Award (MR/X023559/1) and is a co-applicant on grants from PetPlan (S20-924-963), PetSavers (03:20) and PetSavers (MDR 12.22). **Mr Fower** receives salary support from the Morris Animal Foundation (D24FE-810). **Dr Fox** is a co-applicant on a PetPlan grant (S23-1266-1305). **Dr Kaczmarska** has no funding to declare. **Dr Woodhall** is a co-applicant on a PetPlan grant (S23-1266-1305). **Dr Waters** is a co-applicant on grants from PetSavers (MDR 12.22), PetPlan (S23-1266-1305), and the Morris Animal Foundation (D24FE-810). **Dr Handel** is funded by the MRC (MR/X022013/1), NIHR Oxford Health BRC and MyAware and is a co-applicant on a grant from the Morris Animal Foundation (D24FE-810). **Professor Irani** was funded in whole or in part by a senior clinical fellowship from the Medical Research Council [MR/V007173/1], Wellcome Trust Fellowship [104079/Z/14/Z] and by the National Institute for Health Research (NIHR) Oxford Biomedical Research Centre (BRC). **Dr Gutierrez Quintana** holds a grant form CURE Epilepsy. **Dr Eriksson** is supported in part by the National Institute for Health and Care Research University College London Hospitals Biomedical Research Centre funding scheme. **Dr Pakozdy** is a co-applicant on grants from PetPlan (S20-924-963), PetSavers (03:20), PetSavers (MDR 12.22), and the Morris Animal Foundation (D24FE-810).

The funders have not had a role in this study and the views expressed are not necessarily those of the funding bodies. For the purpose of Open Access, the author has applied a CC BY public copyright licence to any Author Accepted Manuscript (AAM) version arising from this submission. The views expressed are those of the author(s) and not necessarily those of the NHS, the NIHR or the Department of Health.

## CRediT authorship contribution statement

**S.N.M. Binks:** Writing – review & editing, Writing – original draft, Visualization, Supervision, Software, Resources, Project administration, Methodology, Investigation, Funding acquisition, Formal analysis, Data curation, Conceptualization. **A.H. Crawford:** Writing – review & editing, Writing – original draft, Visualization, Methodology, Investigation, Funding acquisition, Conceptualization. **E. Ives:** Writing – review & editing, Writing – original draft, Methodology, Investigation, Formal analysis, Conceptualization. **L.J. Davison:** Writing – review & editing, Writing – original draft, Supervision, Funding acquisition. **A. Fower:** Writing – review & editing, Visualization, Project administration, Formal analysis. **H. Fox:** Writing – review & editing, Visualization, Project administration, Formal analysis. **A. Kaczmarska:** Writing – review & editing, Resources. **M. Woodhall:** Writing – review & editing, Supervision, Resources. **P. Waters:** Writing – review & editing, Supervision, Resources. **A.E. Handel:** Writing – review & editing, Writing – original draft, Validation, Supervision, Software, Resources. **S.R. Irani:** Writing – review & editing, Writing – original draft, Supervision, Resources, Funding acquisition. **R. Gutierrez Quintana:** Writing – review & editing, Writing – original draft, Methodology, Investigation, Formal analysis, Conceptualization. **F.A. Chowdhury:** Writing – review & editing, Writing – original draft, Methodology, Investigation, Formal analysis, Conceptualization. **S.H. Eriksson:** Writing – review & editing, Writing – original draft, Investigation, Formal analysis, Conceptualization. **A. Pakozdy:** Writing – review & editing, Writing – original draft, Supervision, Investigation, Funding acquisition, Formal analysis, Conceptualization.

## Declaration of competing interest

The authors declare the following financial interests/personal relationships which may be considered as potential competing interests: **Dr Binks** has received an honorarium from Vetmeduni Wien and is named on a patent application entitled “Diagnostic Strategy to improve specificity of CASPR2 antibody detection” (TBA / BB Ref. JA94536P.GBA). **Dr Handel** has received research funding from UCB-Pharma. **Dr Waters** is a named inventor on patents for antibody assays and has received royalties. He has received honoraria from Biogen Idec, Mereo Biopharma, Retrogenix, UBC, Euroimmun AG, UCB, F. Hoffmann La-Roche, MIAC and Alexion; travel grants from the Guthy-Jackson Charitable Foundation; and research funding from Euroimmun AG. His work in the Autoimmune Neurology Diagnostic Laboratory is supported by the NHS Commissioning service for NMOSD. **Professor Irani** has received honoraria/research support from UCB, Argenx, Immunovant, MedImmun, Roche, Janssen, Cerebral therapeutics, ADC therapeutics, Brain, CSL Behring, and ONO Pharma, and receives licensed royalties on patent application WO/2010/046716 entitled 'Neurological Autoimmune Disorders', and has filed two other patents entitled “Diagnostic method and therapy” (WO2019211633 and US-2021-0071249-A1; PCT application WO202189788A1) and “Biomarkers” (PCT/GB2022/050614 and WO202189788A1). **Dr Eriksson** has received honoraria for educational activities from Eisai, Fidia, Lincoln and UCB pharma, nonrelevant for the current study. **Dr Crawford**, **Dr Ives**, **Professor Davison**, **Mr Fower**, **Dr Fox**, **Dr Kaczmarska**, **Dr Woodhall, Dr Gutierrez Quintana**, **Dr Chowdhury**, and **Dr Pakozdy** declare no competing interests.

## Data Availability

Confidential data, some de-identified data could be made available on request.
