# Supplementary material for: Distinctive seizure signature in the first video case-control study of a naturally-occurring feline autoimmune encephalitis model
Source: Brain Behav Immun. 2025 May;126:289–96. doi: 10.1016/j.bbi.2025.02.018 (PMC12037459; doi:10.1016/j.bbi.2025.02.018)
Supplement: Supplementary Data 1 [file mmc1.docx]

**Supplementary Results Table 1.** Summary of individual included cats.

| ID | Country | Breed | Number of clips | Age/months | Sex | Individual peak onset seizures/day* | LGI1-antibody status | Antibody titre |
| --- | --- | --- | --- | --- | --- | --- | --- | --- |
| Cat 1 | UK | DSH | 1 | 17 | FN | 48 | Positive | 1:40 |
| Cat 2 | UK | DSH | 1 | 96 | FN | 4 | Positive | 1:160 |
| Cat 3 | UK | DSH | 1 | 51 | MN | 20 | Positive | 1:320 |
| Cat 4 | UK | DSH | 1 | 66 | FN | 0.5 | Negative | - |
| Cat 5 | UK | DSH | 1 | 48 | MN | 6 | Positive | 1:80 |
| Cat 6 | UK | DSH | 1 | 27 | FN | 3 | Positive | 1:160 |
| Cat 7 | UK | DSH | 2 | 110 | FN | 5 | Negative | - |
| Cat 8 | Belgium | Exotic | 3 | 31 | MN | 20 | Positive | 1:320 |
| Cat 9 | UK | Bengal Cross | 2 | 53 | FN | 4 | Positive | 1:80 |
| Cat 10 | Italy | DSH | 1 | 66 | MN | Not available | Positive | 1:160 |
| Cat 11 | UK | Bengal | 1 | 4 | ME | 1 | Negative | - |
| Cat 12 | UK | DSH | 1 | 59 | FN | Not available | Positive | 1:20 |
| Cat 13 | Belgium | British Short Hair | 1 | 25 | MN | 24 | Positive | 1:160 |
| Cat 14 | UK | British Short Hair | 1 | 18 | FN | 0.5 | Negative | - |
| Cat 15 | UK | DSH | 1 | 62 | FN | 13 | Positive | 1:320 |
| Cat 16 | UK | DSH | 1 | 4 | FE | 6 | Negative | - |
| Cat 17 | Belgium | Turkish | 3 | 24 | MN | 0.07 | Negative | - |
| Cat 18 | UK | Birman | 1 | 21 | MN | 0.2 | Negative | - |
| Cat 19 | UK | DSH | 2 | 51 | MN | 8 | Positive | 1:80 |
| Cat 20 | UK | DLH | 1 | 18 | MN | 3 | Positive | 1:80 |
| Cat 21 | UK | Ragdoll | 3 | 17 | FN | 7 | Positive | 1:160 |
| Cat 22 | Netherlands | DSH | 1 | 48 | FN | Not available | Negative | - |
| Cat 23 | UK | Bengal | 3 | 48 | MN | 10 | Positive | 1:80 |
| Cat 24 | UK | DSH | 1 | 81 | FN | 6 | Positive | 1:80 |
| *For cats having less than 1 daily seizure at peak, the number of seizures was divided over the time span given e.g. one every two days = 0.5/day | | | | | | | | |

**Abbreviations:** DSH, domestic short hair; DLH, domestic long hair; FN, female neutered; FE, female entire; MN, male neutered; ME male entire

**Supplementary Results Table 2.** Observed features in LGI1-antibody positive compared to LGI1-antibody negative cats, restricted to one clip per cat.

| Feature | LGI1-antibody positive (total observations n =80) | LGI1-antibody negative (total observations n=40) | p-value raw^a^ | | p-value corrected^b^ |
| --- | --- | --- | --- | --- | --- |
| A: Individual observations | | | | | |
| Automatisms | | | | | |
| *Orofacial* | 56 (70%) | 23 (57.5%) | 0.247 | | 1 |
| *Running/pedal* | 6 (7.5%) | 7 (17.5%) | 0.177 | | 1 |
| *Vocalisation* | 21 (26%) | 3 (7.5%) | 0.016 | | 0.263 |
| Autonomic features | | | | | |
| *Mydriasis* | 53 (66%) | 11 (27.5%) | <0.001 | 0.003 | |
| *Respiratory changes* | 14 (17.5%) | 6 (15%) | 0.931 | 1 | |
| *Salivation* | 55 (69%) | 19 (47.5%) | 0.04 | 0.555 | |
| *Urination/defecation* | 1 (1%) | 5 (12.5%) | 0.015 | 0.263 | |
| Awareness | | | | | |
| *Reduced responsiveness* | 56 (70%) | 26 (65%) | 0.729 | 1 | |
| Behavioural features | | | | | |
| *Aggression* | 8 (10%) | 0 (0%) | 0.051 | | 0.660 |
| *Behavioural arrest* | 36 (45%) | 11 (27.5%) | 0.098 | | 1 |
| *Fearful* | 4 (5%) | 1 (2.5%) | 0.664 | | 1 |
| *Restless/searching* | 11 (14%) | 1 (2.5%) | 0.059 | | 0.713 |
| Motor features | | | | | |
| *Circling* | 23 (29%) | 1 (2.5%) | <0.001 | | 0.009 |
| *Head nodding* | 24 (30%) | 9 (22.5%) | 0.515 | | 1 |
| *Head turning/ nodding/version* | 26 (32.5%) | 15 (37.5%) | 0.734 | | 1 |
| *Myoclonus* | 19 (24%) | 11 (27.5%) | 0.823 | | 1 |
| *Sudden jumping* | 11 (14%) | 6 (15%) | 1 | | 1 |
| *Tonic-clonic jerking* | 12 (15%) | 11 (27.5%) | 0.163 | | 1 |
| *Tonic paw extension* | 12 (15%) | 17 (42.5%) | 0.002 | | 0.036 |
| Other | | | | | |
| *GTCS* | 5 (6%) | 8 (20%) | 0.031 | | 0.458 |
| B: Group level observations | | | | | |
| *Any automatism* | 60 (75%) | 25 (62.5%) | 0.227 | | 0.455 |
| *Any autonomic* | 69 (86%) | 24 (60%) | 0.003 | | 0.008 |
| *Any behavioural* | 48 (60%) | 11 (27.5%) | 0.002 | | 0.006 |
| *Any motor* | 60 (75%) | 33 (82.5%) | 0.487 | | 0.487 |
| C: Localisation | | | | | |
| *Temporal lobe* | 53 (66%) | ^c^12 (31%) | <0.001 | | NA |
| ^a^Chisq test (if >5 in all groups) or Fisher’s exact test ^b^Holm corrected for within-group multiple comparisons, corrected p<0.05 taken as significant ^c^One missing observation, total n=39 | | | | | |

**Abbreviations:** GTCS, generalised tonic clonic seizure.

**Supplementary Results Table 3.** ILAE/IVETF classifications in seizure observations from LGI1-antibody positive and LGI1-antibody negative cats.

| **2A: Top level classification** | |
| --- | --- |
| **LGI1-antibody associated seizures** | |
| Focal onset | 86/120 (72%) |
| Unknown onset or no answer given | 33/120 (28%) |
| Generalised onset | 1/120 (<1%) |
| **Seizures not associated with LGI1-antibodies** | |
| Unknown onset | 29/55 (53%) |
| Focal onset | 22/55 (40%) |
| Generalised onset | 3/55 (5%) |
| Non epileptic | 1/55 (2%) |
| **2B: Next level classification** | |
| **LGI1-antibody associated seizures** | |
| Focal motor onset with or without bilateral facial involvement | 65/120 (54%) |
| Focal non motor onset | 23/120 (19%) |
| Unknown non motor onset | 9/120 (8%) |
| Unknown | 5/120 (4%) |
| Unclassified | 4/120 (3%) |
| Unknown motor onset | 4/120 (3%) |
| Focal to bilateral tonic clonic | 4/120 (3%) |
| Generalised tonic clonic | 3/120 (3%) |
| Generalised motor other | 1/120 (1%) |
| Generalised non motor | 1/120 (1%) |
| Missing data | 1/120 (1%) |
| **Seizures not associated with LGI1-antibodies** | |
| Focal motor onset with or without bilateral facial involvement | 19/55 (35%) |
| Unknown onset motor | 13/55 (24%) |
| Unknown | 10/55 (18%) |
| Generalised motor other | 4/55 (7%) |
| Generalised tonic clonic | 2/55 (4%) |
| Focal motor onset | 2/55 (4%) |
| Generalised onset other | 1/55 (2%) |
| Probable jaw pain | 1/55 (2%) |
| Unclassified | 1/55 (2%) |
| Unclassified, possible cardiac | 1/55 (2%) |
| Unknown onset non motor | 1/55 (2%) |

**Supplementary Results 4. Logistic regression calls, calculated odds ratios (exp coef) and significance of models**

**3A: Orofacial automatisms and LGI1-antibody status, at least three expert raters agree on presence of feature**

*Call:*

glm(formula = Antibody_status_binary ~ oral_present, family = binomial,

data = Regression_sumstats3)

Coefficients:

Estimate Std. Error z value Pr(>|z|)

(Intercept) -0.3365 0.5855 -0.575 0.5655

oral_present1 1.8946 0.8034 2.358 0.0184 *

---

Signif. codes: 0 ‘***’ 0.001 ‘**’ 0.01 ‘*’ 0.05 ‘.’ 0.1 ‘ ’ 1

(Dispersion parameter for binomial family taken to be 1)

Null deviance: 43.574 on 34 degrees of freedom

Residual deviance: 37.554 on 33 degrees of freedom

AIC: 41.554

Number of Fisher Scoring iterations: 4

*Odds ratio:*

exp(coef(model))

(Intercept) oral_present1

0.7142857 6.6500000

*Significance of model:*

1-pchisq(43.574-37.554, 34-33)

0.01414465

**3B: Mydriasis and LGI1-antibody status, at least three expert raters agree on presence of feature**

*Call:*

glm(formula = Antibody_status_binary ~ mydriasis_present, family = binomial,

data = Regression_sumstats3)

Coefficients:

Estimate Std. Error z value Pr(>|z|)

(Intercept) -0.3365 0.5855 -0.575 0.5655

mydriasis_present1 1.8946 0.8034 2.358 0.0184 *

---

Signif. codes: 0 ‘***’ 0.001 ‘**’ 0.01 ‘*’ 0.05 ‘.’ 0.1 ‘ ’ 1

(Dispersion parameter for binomial family taken to be 1)

Null deviance: 43.574 on 34 degrees of freedom

Residual deviance: 37.554 on 33 degrees of freedom

AIC: 41.554

Number of Fisher Scoring iterations: 4

*Odds ratio:*

exp(coef(model))

(Intercept) mydriasis_present1

0.7142857 6.6500000

*Significance of model:*

1-pchisq(43.574-37.554, 34-33)

0.01414465

**3C: Temporal lobe origin and LGI1-antibody status, at least three expert raters agree on presence of feature**

*Call:*

glm(formula = Antibody_status_binary ~ temporal_present, family = binomial,

data = Regression_sumstats3)

Coefficients:

Estimate Std. Error z value Pr(>|z|)

(Intercept) -0.1178 0.4859 -0.242 0.8085

temporal_present1 2.1972 0.8936 2.459 0.0139 *

---

Signif. codes: 0 ‘***’ 0.001 ‘**’ 0.01 ‘*’ 0.05 ‘.’ 0.1 ‘ ’ 1

(Dispersion parameter for binomial family taken to be 1)

Null deviance: 43.574 on 34 degrees of freedom

Residual deviance: 36.066 on 33 degrees of freedom

AIC: 40.066

Number of Fisher Scoring iterations: 4

*Odds ratio:*

exp(coef(model))

(Intercept) temporal_present1

0.8888889 9.0000000

*Significance of model:*

1-pchisq(43.574-36.066, 34-33)

0.006142554

**3D: Temporal lobe origin and LGI1-antibody status, at least four expert raters agree on presence of feature**

*Call:*

glm(formula = Antibody_status_binary ~ temporal_present, family = binomial,

data = Regression_sumstats)

Coefficients:

Estimate Std. Error z value Pr(>|z|)

(Intercept) 0.09531 0.43693 0.218 0.8273

temporal_present1 2.46964 1.12598 2.193 0.0283 *

---

Signif. codes: 0 ‘***’ 0.001 ‘**’ 0.01 ‘*’ 0.05 ‘.’ 0.1 ‘ ’ 1

(Dispersion parameter for binomial family taken to be 1)

Null deviance: 43.574 on 34 degrees of freedom

Residual deviance: 36.269 on 33 degrees of freedom

AIC: 40.269

Number of Fisher Scoring iterations: 5

*Odds ratio:*

exp(coef(model))

(Intercept) temporal_present1

1.10000 11.81818

*Significance of model:*

1-pchisq(43.574-36.269, 34-33)

0.0068763

**Supplementary methods: Instructions on how to use the seizure semiology rating tool**

**Semiology (lines 5-31 of spreadsheet)**

1. Semiology features are described and grouped in the far left column (blue sub-heading)
2. For each video, please identify-
   1. If the feature is present in the video – this is a yes/no drop down box (green heading)
   2. The distribution of the feature – there is a menu of choices in the yellow headed column. This includes ‘unknown’ if the feature cannot be observed in the clip, or ‘NA’ (not applicable) if it is a feature without an anatomical distribution
   3. Please enter yes/no for all features so that it is known if the rater felt the feature was present or not

[drop down menus are used to make analysis of data easier at the next project stage]

1. Some description is included in the orange ‘descriptor’ column to help with identifying features that may be more open to interpretation. It is possible to add free text in this box to annotate observed features
2. There is an ‘other’ box (line 31) where features that may be important but not listed in the pre-set boxes can be included

**Classification, localisation and lateralisation (lines 32-37 of spreadsheet)**

1. Based on ILAE position paper – classification of the epilepsies (Scheffer 2017)
2. Categories in the far left column – for each attempt to categorise
   1. Is this possible (yes/no drop down box – if rater does not think this is possible then pick no and do not progress further). There is also an ‘onset not shown’ option if the start of the seizure is not included in the clip
   2. If yes, then further drop down box giving options of classification
3. Some description included in the orange ‘descriptor’ column as not all raters may be familiar with human-based classifications

It is recognised that there will be sometimes when there is difficulty to decide, and that videos only show a snapshot of an episode, in these cases please opt for the **most likely** descriptor based on what can be seen in the clip.

**THANK YOU**
